# Occipital bone and tumor-induced osteomalacia: a rare tumor site for an uncommon paraneoplastic syndrome

**DOI:** 10.1007/s11657-023-01305-y

**Published:** 2023-07-12

**Authors:** Luciano Colangelo, Chiara Sonato, Cristiana Cipriani, Jessica Pepe, Giorgia Farinacci, Biagio Palmisano, Marco Occhiuto, Mara Riminucci, Alessandro Corsi, Salvatore Minisola

**Affiliations:** 1 Department of Clinical, Internal, Anesthesiologic and Cardiovascular Sciences, Sapienza University of Rome, Viale del Policlinico 155, 00161 Rome, Italy; 2 Department of Molecular Medicine, Sapienza University, Rome, Italy

**Keywords:** Tumor-induced osteomalacia, Fibroblast growth factor 23, ^68^ Ga-DOTATATE, Skull base, Occipital bone

## Abstract

**Introduction:**

Tumor-induced osteomalacia (TIO) is an uncommon paraneoplastic syndrome due to the overproduction of fibroblast growth factor 23 (FGF23). It is predominantly caused by mesenchymal tumors and cured upon their complete removal. Non-surgical treatment is an alternative option but limited to specific clinical conditions.

**Methods:**

We report a challenging case of TIO caused by a tumor involving the occipital bone. We also performed a literature review of TIO caused by tumors localized at this site, focusing on clinical findings, treatment, and outcomes.

**Results:**

The patient, a 62-year-old male, presented with a long-lasting history of progressive weakness. Biochemical evaluation revealed severe hypophosphatemia due to low renal tubular reabsorption of phosphate with raised intact FGF23 values. A ^68^ Ga-DOTATATE PET/TC imaging showed a suspicious lesion located in the left occipital bone that MRI and selective venous catheterization confirmed to be the cause of TIO*.* Stereotactic gamma knife radiosurgery was carried out, but unfortunately, the patient died of acute respiratory failure.

To date, only seven additional cases of TIO have been associated to tumors located in the occipital bone. Furthermore, the tumor involved the left side of the occipital bone in all these patients.

**Conclusion:**

The occipital region is a difficult area to access so a multidisciplinary approach for their treatment is required. If anatomical differences could be the basis for the predilection of the left side of the occipital bone, it remains to be clarified.

## Introduction

Tumor-induced osteomalacia (TIO) is an uncommon paraneoplastic syndrome predominantly due to the overproduction of fibroblast growth factor 23 (FGF23) by mesenchymal tumors usually involving bone and soft tissues [1]. An increased number of patients with TIO have been recently reported, possibly reflecting a raised awareness of the disease. A step-by-step clinical approach is fundamental to diagnose TIO and localize the culprit lesion [1, 2]. Non-surgical treatment is an alternative option but is limited to specific clinical conditions, such as inability to locate the tumor, tumor located in sites difficult to be treated surgically, contraindications to surgery, and incomplete previous tumor resection [1, 3–5].

We report a challenging case of TIO located in the occipital bone. Moreover, we also performed a literature review of TIO located in this specific bone focusing on clinical findings, treatment, and outcomes.

## Case presentation

**Fig. 1:**
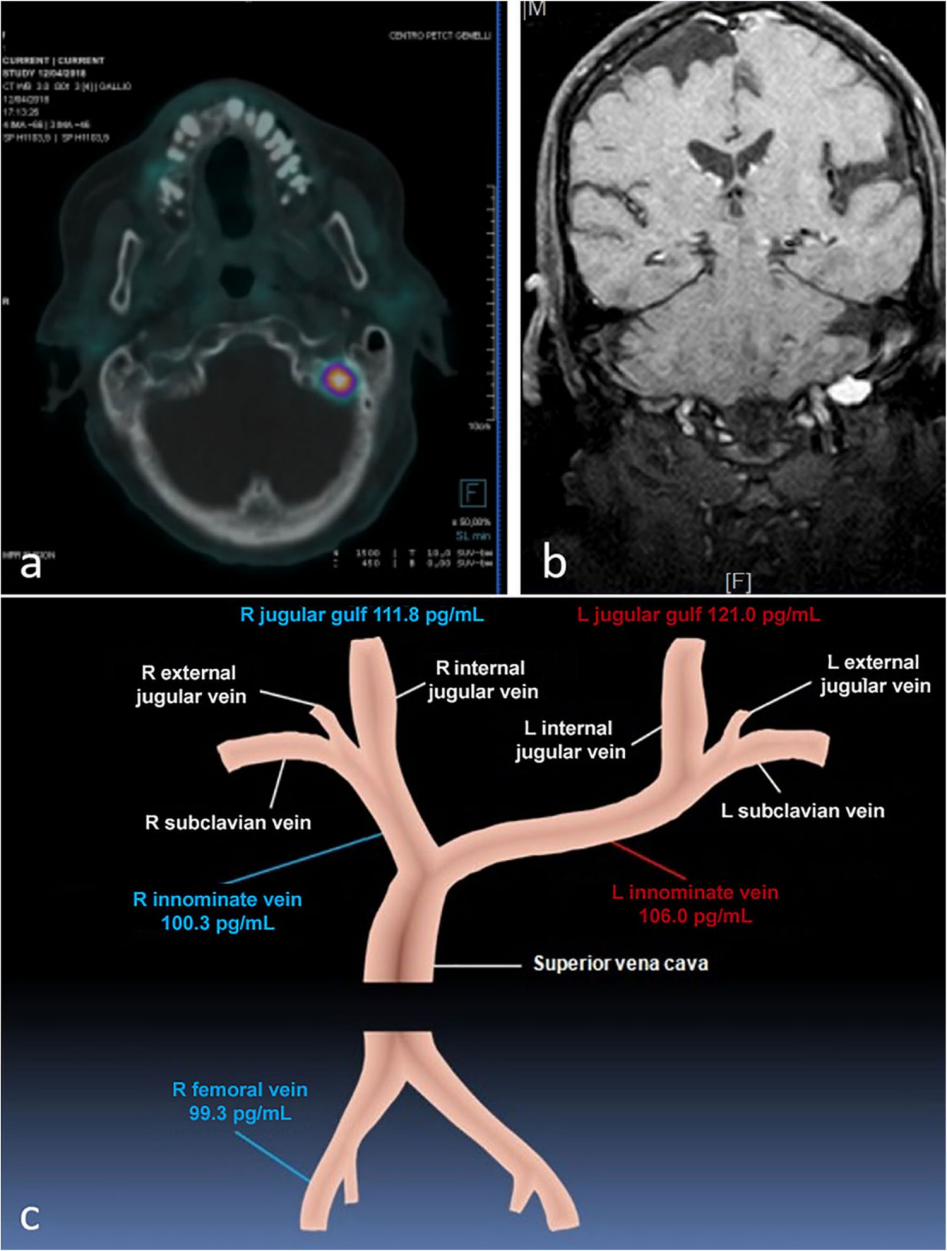
**a** Localization of the tumor in the left occipital bone by ^68^Ga DOTATATE PET/CT and **b** brain MRI are shown respectively. **c** The results of iFGF23 in VBS are illustrated. R: right; L: left

A 62-year-old male presented with an 8 years long-lasting history of weakness. He referred to both hip fractures. Biochemical evaluation at admission showed hypophosphatemia due to low renal tubular reabsorption of phosphate (TmP/GFR: 1.70 mg/dL) and increased serum intact FGF23 value (iFGF23) (190 pg/mL, n.v.: < 95 pg/mL, DiaSorin kit, Stillwater, MN, USA) suggestive of TIO. A ^99m^Tc-labeled octreotide HYNIC-TOC scintigraphy showed increased uptake in the proximal left tibia suggesting it was the culprit site lesion. Even though the targeted MRI was negative, surgery was performed, but the tumor was not histologically detected. In addition, intra-operative assay of serum FGF23 did not show any significant decrease. Then, we performed a ^68^Ga-DOTATE PET/CT, through which a suspicious lesion was detected in the left occipital bone (Fig. 1a). It was also confirmed by brain MRI (Fig. 1b). Venous blood sampling (VBS) for measuring serum iFGF23 on both sides of the brain showed a slight gradient in respect to the contralateral site, suggesting that this was the site of FGF23 overproduction (Fig. 1c). Surgery was refused by the patient (mainly because of the increased risk of mortality, possible irreversible neurological consequences), and stereotactic gamma knife radiosurgery (SGKRS) was carried out. The patient died a few days later owing to acute respiratory failure. Autopsy was not performed.

## Literature review

We searched for all original and review articles in MEDLINE till December 2022. Individual search was carried out using the following terms: “oncogenic osteomalacia,” “tumor induced osteomalacia,” “FGF23,” “skull base,” and “occipital bone.” Syndromic conditions associated with high FGF23 (e.g., fibrous dysplasia/McCune-Albright syndrome) were excluded. Only articles in English were considered. We were able to identify only other seven patients with TIO caused by a tumor located in the occipital bone [6–12].

A clinical synopsis of these patients and of the patient reported here is shown in Table 1. Four patients were males and four were females. Average age was 52.75 ± 5.06 years. Putative duration of the disease was 3.0 ± 2.13 years. All patients complained of at least one typical symptom of chronic hypophosphatemia (i.e., bone pain, weakness, fractures). Mean value of serum phosphate at admission was 1.76 ± 0.26 mg/dL. TmP/GFR values were not available for all patients. Basal circulating FGF23 levels were reported in 6 out of 8 patients. C-terminal assay was used in all cases [8–12] except that reported here in which iFGF23 was measured. As in our case, the tumor was detected in other six patients by functional imaging [7–12]. The suspicious lesion was confirmed by MRI in our case and in other five patients [7–10] and by CT scan in another one [11]. Interestingly, the tumor involved invariably the left side of the occipital bone. In five patients [6, 8–10, 12], the clivus was also involved. In one patient, data on treatment and follow-up were not available [12]. All other previously reported cases were initially treated by surgery. In one of them [9], surgery was performed after angioembolization. Histologically, all excised tumors were consistent with the phosphaturic mesenchymal tumor (PMT) [1]. In three patients [9–11], a postoperative drop of serum FGF23 was documented after surgery. The complete surgical removal of the tumor was obtained in three patients [7, 9, 10], with at least 3 months of follow-up for the two of them. Surgery was unsuccessful in one case [8]. After 4 months of follow-up, a second surgery was performed but again ineffectively. An alternative medical strategy was implemented with phosphate supplements and, for the first time in TIO, with peptide receptor radionuclide therapy (PRRT) ^177^Lu-DOTATATE. Three months after the first cycle, the authors reported improvement of symptoms and lesion’s standard uptake values (SUVs) reduction at ^68^Ga DOTATATE PET/CT. Disease persistence after two surgeries was reported for another case [11]. Then, PRRT^177^Lu-DOTATATE was employed leading to a stable disease after two cycles. Recurrence after 2 years since surgery was reported in one case [6]. The patient was treated with SGKRS followed by subcutaneous octreotide without effect on serum phosphate and FGF23.

**Table 1:** Clinical synopsis of the patients with TIO caused by tumors involving the occipital bone

ID	Author Ref	Gender/age	Putative disease	Location	Localization method	Pi (mg/dL)	FGF23		First treatment	Histology	Persistence or recurrence	Secondary treatment	Duration of follow-up	Outcome
							Pre	Post						
1	[6]	M/45	1 year	Left side and clivus	MRI	1.9	NA	49 pg/mL (nv: 10–50)	Surgery	PMT	Recurrence after 2 years	SGKRS, Phosphorus supplements, 1,25(OH)D and octreotide	NA	NA
2	[7]	M/56	3 years	Left side	SR-PET/CT and MRI	1.9	NA	NA	Surgery	PMT	No	NA	3 months	Recovery
3	[8]	F/53	2 years	Left side and clivus	SR-PET/CT and MRI	1.5	725 RU/mL (nv: < 180)	NA	Surgery	PMT	Persistence after 4 months	Surgery	3 months	Partial recovery
											Persistence after 3 months	Phosphorus supplements, PRRT^177^Lu-DOTATATE		
4	[9]	F/48	2 years	Left side and clivus	SR-PET/CT and MRI	2.1	725 RU/mL (nv: < 180)	155 RU/mL (nv: < 180)	Surgery after angioembolization	PMT	No	NA	NA	Recovery
5	[10]	F/52	3 years	Left side and clivus	SR-PET/CT and MRI	1.6	725 RU/mL (nv: < 180)	150 RU/mL (nv: < 180)	Surgery	PMT	No	NA	3 months	Recovery
6	[11]	F/53	3 years	Left side	^68^ Ga-DOTATATE PET/CT and CT	1.5	725 RU/mL (nv: < 180)	153 RU/mL (nv: < 180)	Surgery	PMT	Persistence	Surgery	13 months	NA
											Second persistence	PRRT^177^Lu-DOTATATE		
7	[12]	F/53	2 years	Left side and clivus	^68^ Ga-DOTATATE PET/CT	1.5	725 RU/mL (nv: < 180)	NA	NA	NA	NA	NA	NA	NA
8	Our case	M/62	8 years	Left side	^68^ Ga-DOTATATE PET/CT, MRI and VBS	2.1	190 pg/mL (nv: < 95)	NA	Radiotherapy	NA	NA	NA	1 month	Death

*SR-PET/TC* somatostatin receptor-based PET/TC, *Pi* phosphorus at admission, *VBS* venous blood sampling, *NA* not available, *Post* after first treatment, *PMT* phosphaturic mesenchymal tumor, *SGKRS* stereotactic gamma knife radiosurgery, *PRRT*^*177*^*Lu-DOTATATE* peptide receptor radionuclide therapy with lutetium-177

## Discussion

The patient reported here is the eighth case of TIO caused by a tumor located in the occipital bone. All patients shared clinical and laboratory findings consistent with TIO caused by tumors located elsewhere and a long delay before final diagnosis, a common finding of patients with TIO [1].

In all surgically treated patients [6–10], the histological diagnosis was consistent with PMT, the most common tumor associated with TIO [1]. It is likely that even in the case reported by Luthra and colleagues [12] and in our case this would have been the diagnosis if the tumor had been excised. Indeed, through ^68^Ga-DOTATE PET/CT, a suspicious area was detected in the left occipital bone. Whole-body ^68^Ga-DOTATATE PET/CT has been reported to have an accuracy for localizing tumors causing TIO greater than that of octreo-SPECT-CT for the higher affinity of ^68^Ga-DOTATATE for somatostatin receptors (in particular type 2) compared to octreotide [1, 13]. However, the expression of somatostatin receptors is not specific for TIO-causing tumors [1, 13]. Indeed, in our case, a histologically proven false-positive increased radiotracer uptake for tumor-causing TIO was detected in the proximal left tibia, likely as a result of osteomalacia-related intramedullary fracture. Furthermore, we were able to confirm tumor localization by VBS (Fig. 1c)*.* Even though a significant threshold to secure the diagnosis has not been established, the detected gradient supported the anatomical origin of FGF23 from the left side. In the patient reported by Hana et al. in which the tumor was located in the anterior skull base, the FGF23 gradient was only 10 pg/mL [14].

Of note, only three patients had a complete recovery after surgery [7, 9, 10]. This percentage is below that reported in the literature [15] emphasizing that occipital bone is a difficult location to approach and for which combined treatments might be necessary. Medical treatments are second-line therapy for TIO patients with undetectable lesions, tumors not completely excised or located in sites difficult to be treated surgically, or with contraindications to surgery [1]. Traditionally, phosphate salts and active vitamin D metabolites are employed. Calcimimetics have been rarely used. Burosumab (an anti-FGF23 monoclonal antibody) has been recently approved for the treatment of patients with TIO based on the results obtained in two clinical trials [3, 4]. However, in these studies, the results were not entirely satisfactory. For example, mean serum phosphate was barely in the normal range, fractures persisted after 2 years of treatment, new fractures developed, and the effect on pain was absent in one study [3] and moderate in the other [4]. If these findings reflect the need to better define the appropriate dosing regimen [5] and/or the expression (and secretion) by tumor cells of other phosphatonins (e.g., MEPE, FGF7, sFRP-4) [1, 5, 16–18], it remains to be established. Interestingly, in two cases of TIO caused by tumors located in the occipital bone, PRRT^177^Lu-DOTATATE was used [8, 11]. Even though some short-term benefits were observed in both cases, further studies are needed to evaluate the safety and efficacy of PRRT^177^Lu-DOTATATE on a long-term period.

Finally, the finding is of interest in that in all patients, the tumor was localized in the left side of the occipital bone, and in five of them, the clivus was also involved. The existence of anatomical differences between the right and left sides of the occipital bone is well established. For example, mean values of condylar width and sagittal angle measurements were found significantly higher on the left side compared to the right one [19]. However, how these differences can reconcile with the development of the tumor remains questionable. The contribution of developmental anomalies involving the occipital bone, as in other more common, albeit rare, pathologic conditions occurring at this site (i.e., notochordal remnants/inclusions and chordoma) [20], seems unlikely. Indeed, PMTs fail to show any skeletal and soft tissue site–specific distribution having been reported virtually everywhere [1, 2].

In conclusion, we presented a new case of TIO caused by a tumor involving the occipital bone and a review of the pertinent literature regarding TIO associated with tumors occurring at this site. A meticulous patient analysis was performed to detail relevant clinical aspects. Major limitations are the retrospective nature of the study and the lack of important laboratory data such as pre- and/or post-treatment serum FGF23 levels and histological diagnosis in some cases. Overall, our analysis indicates that the occipital bone represents a challenging location for TIO-causing tumors for which a multidisciplinary approach is required to obtain the final cure of the patient.

## Data Availability

The data sets used and/or analyzed during the current study are available from the corresponding author on reasonable request.
